# Oral immunotherapy with raw egg: immunological data at 6 and 18 months

**DOI:** 10.1186/2045-7022-3-S3-P18

**Published:** 2013-07-25

**Authors:** PM Ojeda, I Ojeda, G Rubio, F Pineda

**Affiliations:** 1 Allergy, Clínica de Asma y Alergia Dres. Ojeda, Madrid, Spain; 2 Research, Diater Laboratories, Madrid, Spain

## Background

The efficacy, tolerability and immunological results of a SOTI regime with liquid pasteurized egg (SOTI-LPE) (both yolk and white) in 36 egg allergic children have already been published [1]. We present the immunological data at 6 (visit R1) and 18 months (visit R2) after completing SOTI.

## Methods

By protocol skin prick testing (SPT) with egg white (EW), yolk (EY), ovoalbumin (OVA), and ovomucoid (OVM); specific IgE against white, yolk, OVA, and OVM, and specific IgG4 against OVA and OVM were performed at 6 and 18 months after completing the SOTI regime. The results were compared with those of the inclusion visit (B0).

## Results

SPT showed a trend towards a decrease in the wheal surface area (mean in mm^2^ B0 vs mean R1, p value; vs mean R2, p value): EW 97.5 vs 38.0, p<0.0001; vs 36.3, p<0.001; EY 33.5 vs 12.4 p<0.0001; vs 12.3, p=0.003; OVA 55.6 vs 19.1 p<0.0001; vs 17.3, p<0.001; OVM 109.6 vs 56.9, p=0.005; vs 44.8, p=0.000.

Mean sIgE values tended to increase at R1 and then decrease at R2 (IgE kU/L B0 vs R1, p value; vs R2, p value): EW 12.2 vs 28.8, p<0.0001; vs 10.6, p=0.322; EY 9.4 vs 7.1, p<0.0001; vs 7.6, p=0.069; OVA 9,4 vs 11.6, p=0.002; vs 8.7, p=0.95; OVM 10.9 vs 24.2, p<0.0001; vs 8.9, p=0.015.

Mean sIgG4 values significantly increased (IgG4 mg/L B0 vs R1, p value; vs R2, p value): OVA 1.2 vs 16.9, p<0.001; vs 18.9, p<0.001; OVM 1.1 vs 5.0, p<0.001; vs 8.1, p<0.001. IgG4 increases were also seen in patients not acquiring clinical tolerance to LPE.

## Conclusion

Global immunological results indicate significant changes suggesting the stimulation of immunological tolerance pathways in egg allergic children treated with SOTI-LPE: a decrease in skin reactivity and increase of sIgG4 antibodies. As a whole, IgE antibodies did not significantly change at 18 months as compared to baseline.

## Disclosure of interest

None declared.
